# Exploratory analyses of treatment subgroup interaction by PD-L1 status and according to PD-L1 expression in the JAVELIN Bladder 100 trial

**DOI:** 10.1007/s12094-023-03358-4

**Published:** 2023-12-15

**Authors:** Miguel Ángel Climent, Carlos Álvarez, Rafael Morales, Pablo Maroto, Alejo Rodríguez-Vida, María José Méndez-Vidal, Xavier García del Muro, Javier Puente, Nuria Láinez, Sergio Vázquez, Daniel Castellano, Carmen Gómez Lang, Jing Wang, Alessandra di Pietro, Craig Davis, Belén Sanz-Castillo, M. Victoria Bolós, Begoña P. Valderrama

**Affiliations:** 1 Valencian Institute of Oncology, Valencia, Spain; 2 Hospital Universitario Central de Asturias, Oviedo, Spain; 3 Vall d’Hebron University Hospital, Barcelona, Spain; 4 Hospital Universitari de La Santa Creu I Sant Pau, Barcelona, Spain; 5 Hospital del Mar-CIBERONC, IMIM Research Institute, Barcelona, Spain; 6 Maimonides Institute for Biomedical Research of Cordoba (IMIBIC), Reina Sofia University Hospital (HURS), Cordoba, Spain; 7 Catalan Institute of Oncology, Barcelona, Spain; 8 Hospital Universitario Clínico San Carlos, Madrid, Spain; 9 University Hospital of Navarra, Pamplona, Spain; 10 Hospital Universitario Lucus Augusti, Lugo, Spain; 11 Hospital 12 de Octubre, Madrid, Spain; 12 Merck S.L.U., an Affiliate of Merck KGaA, Madrid, Spain; 13 Pfizer, Cambridge, MA USA; 14 Pfizer Srl, Milan, Italy; 15 Pfizer Translational Oncology, La Jolla, CA USA; 16 Pfizer Oncology, Madrid, Spain; 17 Virgen del Rocío University Hospital, Seville, Spain

**Keywords:** Advanced urothelial carcinoma, PD-L1, Avelumab, Maintenance treatment, Interaction test

## Abstract

**Purpose:**

Post hoc analysis of the JAVELIN Bladder 100 trial of avelumab maintenance in locally advanced/metastatic urothelial carcinoma (la/mUC) to determine the interaction by programmed death ligand 1 (PD-L1) status for overall survival (OS), and additional analyses of survival per a different PD-L1 expression cutoff of ≥ 1% in tumor cells or immune cells (TC/IC).

**Methods:**

JAVELIN Bladder 100 data were used for the analysis of the interaction by PD-L1 status (per cutoff used in the trial) for OS and, additionally, OS and progression-free survival (PFS) analyses per a different ≥ 1% TC/IC PD-L1 expression cutoff (Ventana SP263 assay).

**Results:**

No significant interaction between treatment and PD-L1 status was observed for OS. Clinically meaningful and robust survival data were observed in favor of avelumab using the different ≥ 1% TC/IC PD-L1 expression cutoff.

**Conclusions:**

These results demonstrate the benefit of avelumab maintenance in la/mUC regardless of PD-L1 expression, consistent with approved labels.

## Introduction

The phase 3 randomized JAVELIN Bladder 100 trial enrolled patients with locally advanced/metastatic urothelial carcinoma (la/mUC) that had not progressed with first-line (1L) platinum-based chemotherapy [1]. Overall survival (OS, primary endpoint) and progression-free survival (PFS) were significantly prolonged with avelumab 1L maintenance plus best supportive care (BSC) vs BSC alone in both primary populations: the overall population (all randomized patients) and the programmed death ligand 1 (PD-L1)–positive population [1]. Based on these results, avelumab 1L maintenance was approved for patients with la/mUC that had not progressed with 1L platinum-based chemotherapy [2], regardless of tumor PD-L1 expression; it is now recommended as the standard of care in international treatment guidelines [3, 4]. Although in JAVELIN Bladder 100 the magnitude of the benefit in terms of OS was higher among patients with PD-L1-positive tumor cells (TC) or immune cells (IC) than among patients with PD-L1-negative tumors, the study was not powered to compare outcomes with avelumab in the PD-L1–negative subgroup. However, a non-statistically significant increase in OS was also observed in favor of avelumab plus BSC in patients with PD-L1–negative tumors in the primary analysis (hazard ratio [HR], 0.85; 95% CI, 0.62–1.18) [1]. These results were also confirmed with longer follow-up (≥ 2 years in all patients) (HR, 0.83; 95% CI, 0.63–1.10) [5, 6]. Furthermore, higher 12-month PFS rates (21.6% vs 7.1%) and objective response rates (ORRs; 5.8% vs 0.8%) were observed with avelumab plus BSC vs BSC alone in patients with PD-L1–negative tumors [1].

Exploratory biomarker analyses of OS in JAVELIN Bladder 100 suggested that although established biomarkers such as PD-L1 and tumor mutational burden (TMB) showed some predictive value, each biomarker alone missed important subgroups of patients who could potentially benefit from therapy [7]. A HR of 0.44 (95% CI, 0.251–0.768) was observed in patients with PD-L1–negative tumors and a high TMB [7]. Moreover, an analysis of patients who had received avelumab for ≥ 12 months showed that 33.1% (39/118) had a PD-L1–negative tumor [6]. Overall, available evidence from JAVELIN Bladder 100 indicates that avelumab provides benefit regardless of tumor PD-L1 status [1, 2, 6, 7].

We report 2 exploratory analyses of the efficacy of avelumab by tumor PD-L1 expression in patients with la/mUC enrolled in JAVELIN Bladder 100. The objective was to determine if there was an interaction between avelumab and PD-L1 status (positive, negative, or unknown) for OS based on the standard definition of PD-L1 expression used in the trial and validated by the Ventana SP263 PD-L1 assay manufacturer (PD-L1-positive status if at least one of the following three criteria were met: at least 25% of TC stained for PD-L1, at least 25% of IC stained for PD-L1 if more than 1% of the tumor area contained IC, or 100% of IC stained for PD-L1 if no more than 1% of the tumor area contained IC). Additionally, we conducted a post hoc analysis for efficacy reclassifying the trial samples with a different PD-L1 expression cutoff of ≥ 1% TC or IC, which identified more PD-L1–positive tumors than the standard definition.

## Materials and methods

Detailed methodology of JAVELIN Bladder 100 (NCT02603432) has been described previously [1]. Eligible patients had la/mUC and were progression-free after 4–6 cycles of 1L chemotherapy (cisplatin and/or carboplatin plus gemcitabine). Patients were randomized (1:1) to avelumab plus BSC (n = 350) or BSC alone (n = 350). Treatment continued until patient withdrawal, confirmed disease progression, unacceptable toxicity, or other criteria for discontinuation occurred. In the primary analysis, PD-L1–positive status was defined as meeting 1 of the following criteria: expression in ≥ 25% of TCs, expression in ≥ 25% of tumor-associated ICs if the percentage of ICs was > 1%, or expression in 100% of tumor-associated ICs if the percentage of ICs was ≤ 1% (per the SP263 assay label) [1].

To assess the heterogeneity of treatment effect for OS across PD-L1 status (positive, negative, or unknown), a Cox regression model was fitted with OS as the dependent variable and PD-L1 status, treatment, and treatment-by-PD-L1 interaction as explanatory variables. A p value for the interaction test (Wald χ^2^) was provided together with the adjusted OS effect for PD-L1 status. P values are not adjusted for multiplicity. The analysis was conducted with data with a median follow-up of 21 months (data cutoff: October 21, 2019) and 38 months (data cutoff: June 4, 2021).

To conduct post hoc analyses determining OS and PFS using different PD-L1 expression cutoffs, patients were classified as having tumors with < 1% TC/IC (0% of TCs with any membrane staining above background level and 0% of tumor-associated ICs with staining of any intensity above background level) or ≥ 1% TC/IC (≥ 1% of TCs or tumor-associated ICs with staining above background level). PFS was established per RECIST version 1.1 by blinded independent central review (BICR) or investigator assessment.

Given the exploratory nature of these analyses, no definitive conclusion can be drawn from the analyses, and p values should only be viewed as descriptive.

## Results

### Interaction test of treatment by tumor PD-L1 status

In JAVELIN Bladder 100, 358 patients (51.1%) had PD-L1–positive tumors, 270 patients (38.6%) had negative tumors, and 72 patients (10.3%) had unknown tumor status per the SP263 assay label. The interaction test results suggest that no significant interaction (p > 0.05) between treatment and PD-L1 status for OS was observed after a median follow-up of 21 months (Table 1) or 38 months (Table 2).

**Table 1:** Interaction test for OS of treatment by patient’s tumor PD-L1 status after a median follow-up of 21 months

Covariate	Model	Avelumab + BSC (n = 350)		BSC alone (n = 350)		Parameter estimate	Standard error	p value^a^	HR (95% CI)^b^
		n	Events, n (%)	n	Events, n (%)				
PD-L1 status at baseline^c^	Negative	139	76 (54.7)	132	72 (54.5)	0.209	0.1618	0.1968	–
	Unknown	22	8 (36.4)	49	25 (51.0)	0.170	0.2287	0.4576	–
	Positive	189	61 (32.3)	169	82 (48.5)	–	–	–	–
	Treatment^a^ negative	–	–	–	–	0.413	0.2361	**0.0803**	–
	Treatment^a^ unknown	–	–	–	–	0.182	0.4403	**0.6792**	–
	Treatment	–	–	–	–	− 0.570	0.1693	0.0008	–
	Adjusted treatment effect for negative	–	–	–	–	–	–	–	0.85 (0.619–1.181)
	Adjusted treatment effect for unknown	–	–	–	–	–	–	–	0.68 (0.306–1.505)
	Adjusted treatment effect for positive	–	–	–	–	–	–	–	0.57 (0.406–0.788)

N is the number of patients with nonmissing values of the covariate in the associated subgroup and treatment group and is the denominator used to calculate percentages. Models were run separately for each covariate, including treatment effect in the model. Data cutoff: October 21, 2019

BSC, best supportive care; HR, hazard ratio; OS, overall survival; PD-L1, programmed death ligand 1

^a^The 2-sided p value for the parameter estimate is based on the Wald χ^2^ test

^b^Based on the Cox proportional hazards model. An HR < 1 indicates better outcomes with avelumab + BSC vs BSC alone

^c^For categorical variables, the last category listed is treated as the reference and has a value of 0 in the model

**Table 2:** Interaction test for OS of treatment by patient’s tumor PD-L1 status after a median follow-up of **38 months**

Covariate	Model	Avelumab + BSC (n = 350)		BSC (n = 350)		Parameter estimate	Standard error	p value^a^	HR (95% CI)^b^
		n	Events, n (%)	n	Events, n (%)				
PD-L1 status at baseline^c^	Negative	139	101 (72.7)	131	100 (76.3)	0.326	0.1391	0.0191	–
	Unknown	22	12 (54.5)	50	29 (58.0)	0.119	0.2095	0.5715	–
	Positive	189	102 (54.0)	169	108 (63.9)	–	–	–	–
	Treatment^a^ negative	–	–	–	–	0.184	0.1976	**0.3526**	–
	Treatment^a^ unknown	–	–	–	–	0.140	0.3702	**0.7055**	–
	Treatment	–	–	–	–	− 0.366	0.1383	0.0081	–
	Adjusted treatment effect for negative	–	–	–	–	–	–	–	0.83 (0.632–1.099)
	Adjusted treatment effect for unknown	–	–	–	–	–	–	–	0.80 (0.407–1.564)
	Adjusted treatment effect for positive	–	–	–	–	–	–	–	0.69 (0.529–0.909)

N is the number of patients with nonmissing values of the covariate in the associated subgroup and treatment group and is the denominator used to calculate percentages. Models were run separately for each covariate, including treatment effect in the model. Data cutoff: June 4, 2021

BSC, best supportive care; HR, hazard ratio; OS, overall survival; PD-L1, programmed death ligand 1

^a^The 2-sided p value for the parameter estimate is based on the Wald χ^2^ test

^b^Based on the Cox proportional hazards model. An HR < 1 indicates better outcomes with avelumab + BSC vs BSC alone

^c^For categorical variables, the last category listed is treated as the reference and has a value of 0 in the model

### Post hocanalysis of OS and PFS by different PD-L1 expression cutoffs

**Fig. 1:**
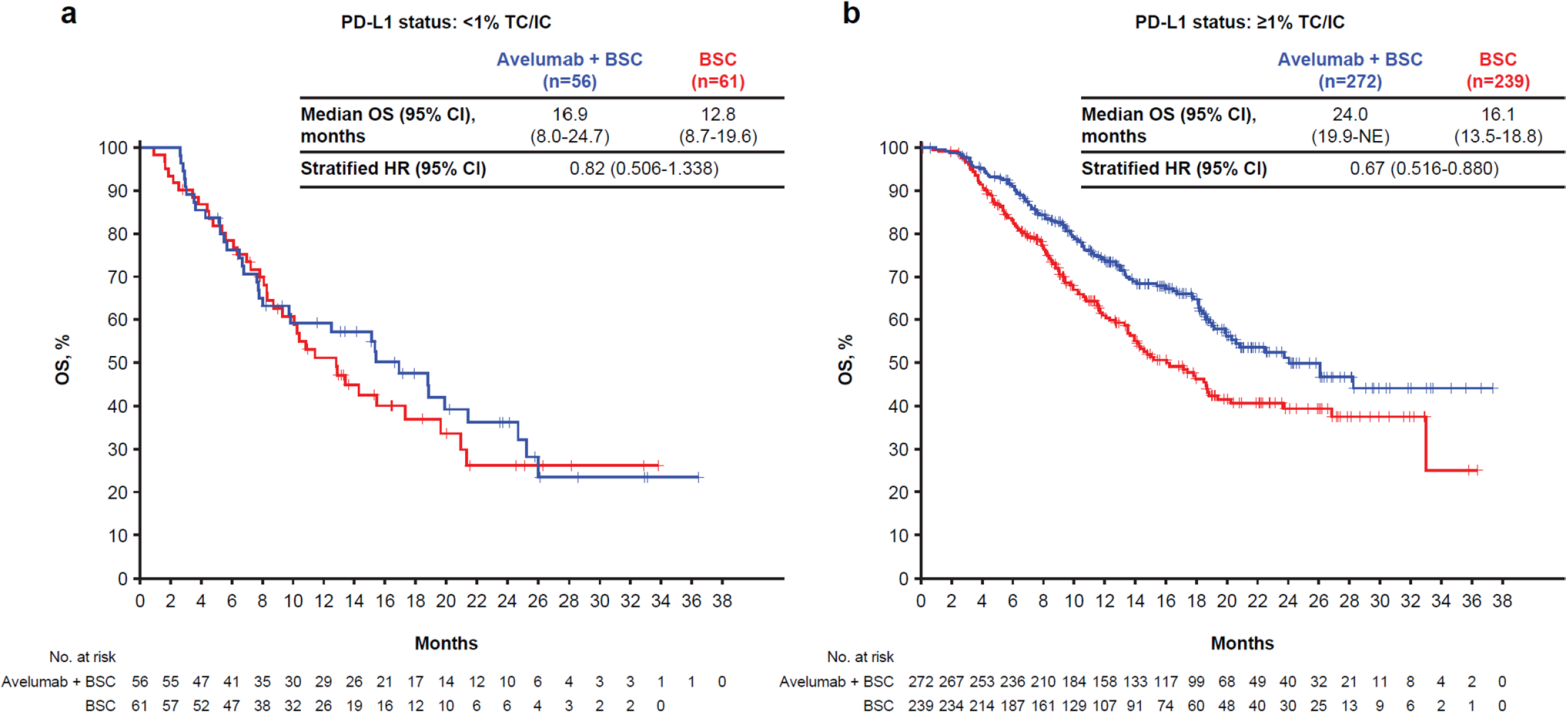
OS per treatment and PD-L1 expression status. **a** Patients with < 1% TC/IC tumors; **b** Patients with ≥ 1% TC/IC tumors. Data cutoff: October 21, 2019. Data snapshot: November 21, 2019. BSC, best supportive care; HR, hazard ratio; IC, immune cell; NE, not estimable; OS, overall survival; PD-L1, programmed death ligand 1; TC, tumor cell

Using the ≥ 1% TC/IC cutoff, 511 of 628 evaluable patients (81.4%) were classified as having a PD-L1 ≥ 1% tumor. A significant improvement in OS was observed with avelumab plus BSC vs BSC alone in the PD-L1 ≥ 1% population (HR, 0.67; 95% CI, 0.516–0.888; median, 24.0 vs 16.1 months) (Fig. 1).

**Fig. 2:**
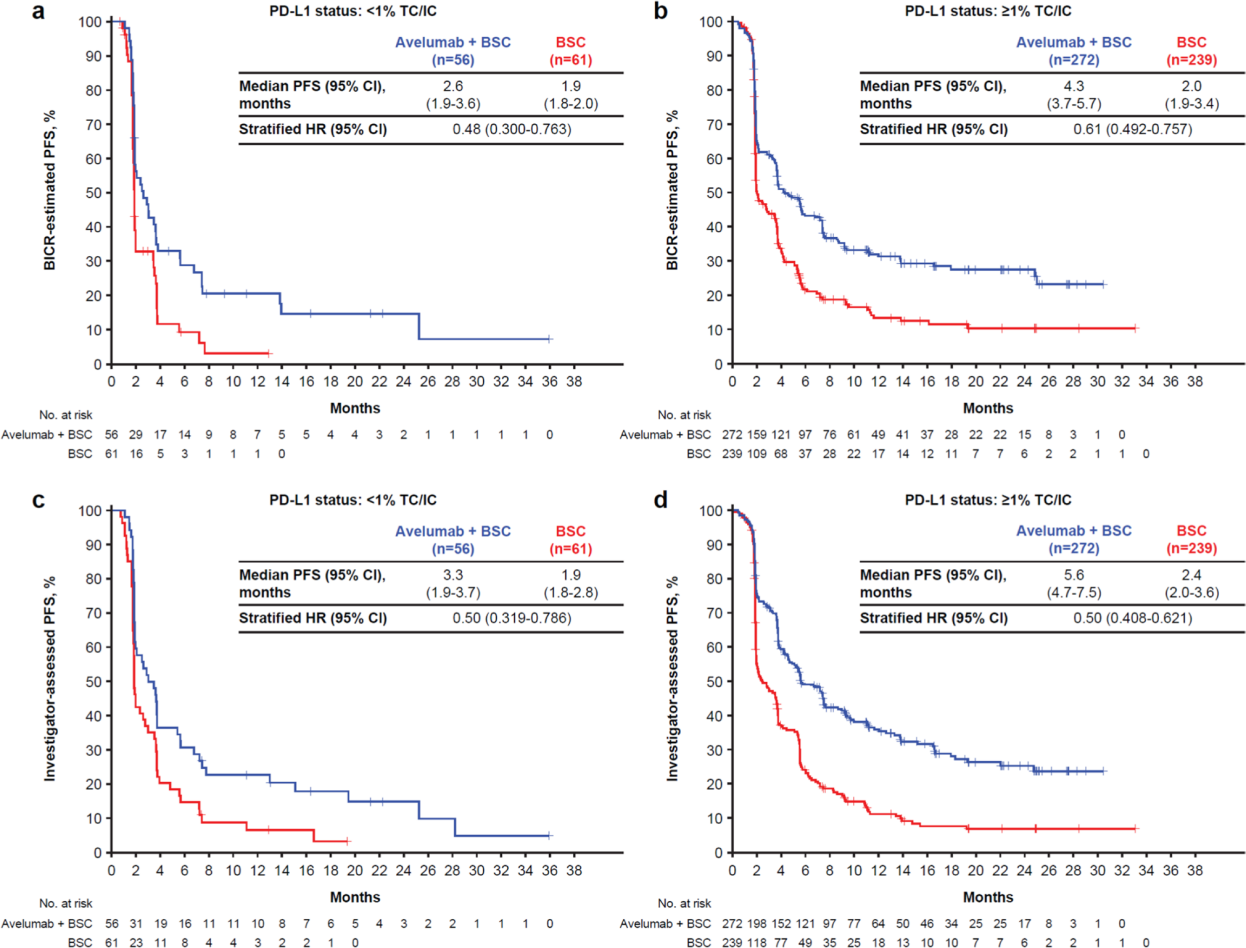
PFS per treatment and PD-L1 expression status. **a** BICR-estimated PFS in patients with < 1% TC/IC tumors; **b** BICR-estimated PFS in patients with ≥ 1% TC/IC tumors; **c** Investigator-assessed PFS in patients with < 1% TC/IC tumors; **d** Investigator-assessed PFS in patients with ≥ 1% TC/IC tumors. Data cutoff: October 21, 2019. Data snapshot: November 21, 2019. BICR, blinded independent central review; BSC, best supportive care; HR, hazard ratio; IC, immune cell; PD-L1, programmed death ligand 1; PFS, progression-free survival; TC, tumor cell

Similarly, significant improvements in PFS were observed with avelumab plus BSC vs BSC alone in the PD-L1 ≥ 1% population by BICR (HR, 0.50; 95% CI, 0.408–0.621; median, 5.6 vs 2.4 months) or investigator assessment (HR, 0.61; 95% CI, 0.492–0.757; median, 4.3 vs 2.0 months) (Fig. 2).

## Discussion

We present results of 2 exploratory descriptive analyses of the efficacy of avelumab 1L maintenance plus BSC vs BSC alone by tumor PD-L1 status in JAVELIN Bladder 100. First, the results of the interaction test between avelumab treatment and PD-L1 status were nonsignificant for OS. This analysis demonstrates that avelumab maintenance benefits all populations regardless of tumor PD-L1 status. Secondly, post hoc analyses for efficacy according to a differently defined PD-L1 expression cutoffs (≥ 1% TC/IC) identified 81.4% of evaluable patients as having PD-L1–positive tumors. Clinically meaningful and robust OS and PFS benefits in favor of avelumab were seen, consistent with the results of the primary analysis of JAVELIN Bladder 100 using the PD-L1 expression cutoff from the SP263 assay label [1].

Despite the approval of several immune checkpoint inhibitors for the treatment of UC, predictive biomarkers are still lacking. Different approaches are necessary because PD-L1 is the only approved biomarker for UC and its predictive value has been inconsistent [8, 9]. In patients with platinum-refractory la/mUC, higher levels of PD-L1 in infiltrating ICs correlated with better ORRs in early studies with atezolizumab, but in the subsequent phase 3 IMvigor211 trial, high PD-L1 expression did not predict greater benefit from atezolizumab vs chemotherapy [8, 10]. However, an exploratory analysis of the phase 3 IMvigor130 trial showed prolonged OS with atezolizumab monotherapy vs chemotherapy in cisplatin-ineligible patients with PD-L1–positive tumors [11]. Likewise, the initial development of pembrolizumab focused on patients with la/mUC with tumors expressing PD-L1 in ≥ 1% of TCs or stroma [12], but the pivotal phase 3 KEYNOTE-045 trial in platinum-refractory patients with la/mUC showed that the OS benefit with pembrolizumab vs chemotherapy was independent of PD-L1 status [13]. Furthermore, an exploratory analysis of the phase 3 KEYNOTE-361 trial suggested that PD-L1 combined positive score did not predict better outcomes with pembrolizumab in cisplatin-ineligible patients [14]. Although both atezolizumab and pembrolizumab were initially approved in the 1L setting in cisplatin-ineligible patients with PD-L1–positive tumors, the US Food and Drug Administration (FDA) later modified the label for pembrolizumab, limiting it to platinum-ineligible patients independent of PD-L1 expression, and the atezolizumab indication was withdrawn [8].

An exploratory biomarker analysis of JAVELIN Bladder 100 suggested that individual biomarkers did not comprehensively identify patients who could benefit from avelumab [7]. Although established biomarkers such as PD-L1 and TMB showed some predictive value, each biomarker alone missed important subgroups of patients who could potentially benefit from therapy [7]. An exploratory analysis of patients with long-term avelumab treatment (≥ 12 months) in JAVELIN Bladder 100 showed that one-third of these patients had PD-L1–negative tumors [6].

In the post hoc analysis of JAVELIN Bladder 100 presented here, a lower number of patients were classified as having PD-L1–negative tumors using a different cutoff for the Ventana SP263 assay (≥ 1% TC/IC) vs the cutoff of the SP263 assay label used in the trial’s primary analysis (18.6% vs 38.6%). Overall, the results of efficacy using the ≥ 1% TC/IC PD-L1 expression cutoff were consistent with those of analyses using the previous PD-L1 expression cutoff from the SP263 assay label [1]. Although these PD-L1 expression definitions have not been analytically validated for UC by the SP263 manufacturer, these data also suggest that a PD-L1 expression cutoff of ≥ 1% TC/IC may be more consistent with the label of avelumab and useful if patient selection based on PD-L1 status was required with the Ventana SP263 assay or similar [15]. Based on evidence from JAVELIN Bladder 100, it would not be justified to exclude patients with PD-L1–negative tumors from avelumab 1L maintenance treatment since these patients would not have access to better therapeutic alternatives.

Although the exploratory descriptive analyses presented here should be interpreted with caution, this additional evidence from JAVELIN Bladder 100 supports the conclusion that avelumab 1L maintenance provides a clinical benefit regardless of PD-L1 status, consistent with the European Medicines Agency and FDA labels [2].

## Data Availability

The authors will provide data included in the present research upon request.
